# Post-Graduate Assistance

**Published:** 1920-04-03

**Authors:** 


### The Hospital

The Workers' Newspaper of Administrative Medicine and Institutional Life, Administration, National Insurance and Health.

No. 1765, Vol. LXVIII. SATURDAY, APRIL 3, 1920. PRICE TWOPENCE

### POST-GRADUATE ASSISTANCE.

Both the hospital and the university worlds should give whole-hearted support to the plea for post-graduate financial support from the Government for those young medical officers who were mobilised immediately after taking their Conjoint diplomas, and, having meanwhile passed their earlier university examinations, now find themselves greatly handicapped in completing the senior course and going up for the M.B. degree.

The matter was brought prominently forward by Sir Wilmot Herringham in a recent letter to *The Times*, in which, as a result of first-hand experience in France, he explains that a young man, after the average three years of active war service, has inevitably forgotten a large part of the knowledge necessary to success in an M.B. examination, and needs, as a minimum, six months of hard work in which to achieve the required standard. Under existing circumstances the expense of so long a non-earning period is proving in many, perhaps in the majority of, cases too great to be borne, and it is not surprising that, with the writer of the letter, one hears on every hand of men reluctantly abandoning and postponing their university finals.

The unsatisfactory nature of this development must be realised from two distinct and separate standpoints. We join our medical contemporaries in urging that these young medical officers have deserved well of their country and that something ought in justice to be done for those men who find that post-war financial difficulties tend to rob them of the educational opportunity they would otherwise have had. Thus Sir Wilmot Herringham makes the timely suggestion that on demobilisation they should, in suitable cases, receive a grant towards the finishing of their medical education. This, however, 'is but a part of the story. As we write there are a considerable number of civilian medical officers still compulsorily, perhaps necessarily, retained on duty with the R.A.M.C. in both the European and Eastern areas. It may be actually true that reliefs *cannot* be found for them, but it is certainly a fact that when these men do return their right to opportunity for reconstructing and recommencing a broken educational career will be very obvious. Representation is being made to the War Office that young R.A.M.C. officers detained in the East should, whenever possible, be recalled in order to continue their studies; but, deserved though such action would be, we feel that the next step should also be taken and financial assistance provided for those in need of it.

The country owes its consideration to the younger men with Service records in the Navy and Army, and although the former shortage of resident medical officers in civil hospitals—a matter to which attention was ultimately given in the early part of last year—has now been to a great extent remedied, it is still to be pointed out that, quite apart from the value of a university degree to a doctor himself, there is the immediate and potential worth of the more highly trained men in every matter concerning national health.

We read that the Australian Government has already approved of a scheme which provides officers of the A.A.M.C. with opportunity to obtain post-graduate instruction before returning to their own country. Briefly, it may be explained that during his period of study the officer will receive full pay and allowances, together with 5s. a day subsistence allowance and approved tuition fees, besides retaining the honorarium from any post which he might hold. It is true that there are certain differences in the main circumstances of this plan and those which we must face at home, but the principles and the meanings therein which concern both the health of the respective countries and their appreciation of war work done are essentially the same. Australia gives us an example to be followed, and we would draw attention to the hardship, the injustice, and the shortsightedness which accompany a ruling that a student may be helped to qualify but that a qualified man cannot be assisted to obtain a higher degree. During the emergencies of war and a need, fancied or real, for every possible medical man to be in the fighting areas this decision was relatively correct, but nowadays it is distinctly otherwise.

